# Examining the Predictors of Life Satisfaction in College Students With Intellectual Disability

**DOI:** 10.1111/jir.70090

**Published:** 2026-02-18

**Authors:** Emily K. Van Gaasbeek, Marc J. Tassé

**Affiliations:** ^1^ Nisonger Center The Ohio State University Columbus Ohio USA

## Abstract

**Background:**

Young adults with intellectual disability (ID) are increasingly attending college through inclusive post‐secondary education (IPSE) programmes. However, little is known about their psychological experiences at college. The current study aimed to examine the role of belongingness, anxiety and depressive symptoms in predicting life satisfaction for college students with ID enrolled in IPSE programmes at 4‐year American colleges and universities.

**Method:**

College students with ID (*n* = 129) from 21 IPSE programmes across the United States completed an online survey reporting on mental health symptoms, belongingness and life satisfaction. Programme staff (*n* = 21) from each of the IPSE programmes also completed an online survey providing information about their programmes.

**Results:**

Results of the study indicated that there were significant differences in life satisfaction and level of anxiety based on gender, residential status and access to mental health services. A hierarchical regression analysis revealed that belongingness was significantly associated with life satisfaction and accounted for 12.5% additional variance in life satisfaction, above and beyond the variance accounted for by race, ethnicity, residential status and mental health symptoms.

**Conclusion:**

This study provides information on the well‐being of college students with ID attending IPSE programmes, indicating high rates of mental health symptoms along with the role played by their sense of belonging in relation to their reported life satisfaction.

## 1 Background

Adults with intellectual disability (ID) have historically been excluded from college, due to admissions requirements of a regular high school diploma and high grade point averages. The US Higher Education Opportunity Act (HEOA), a federal law passed in 2008, significantly increased opportunities for adults with ID to attend college through inclusive post‐secondary education (IPSE) programmes. The extant literature has reported positive effects of attending IPSE programmes (Butler et al. 2016; Lee et al. 2021; Prohn et al. 2018; Ryan et al. 2019; Spencer et al. 2021). However, most research on IPSE programmes has used data from third‐party respondents such as programme staff or parents. When the perspectives of students are solicited, the data collected are typically qualitative in nature, limiting the ability to make comparisons or generalisations (Berg et al. 2017; Hendrickson et al. 2013; Jones and Goble 2012; Paiewonsky 2011; Rosencrans et al. 2021; Whirley et al. 2020). Given that most research on neurotypical college students has relied on self‐report data, failing to use self‐report measures among college students with ID limits the ability to fully understand their experiences and compare with other college students.

Research has suggested a growing prevalence of mental health disorders among college students. Nationwide surveys have estimated more than 60% of college students report at least one mental health disorder (American College Health Association 2022; Lipson et al. 2022). Anxiety and depressive symptoms are the most prevalent among neurotypical college students. A recent nationwide survey of college students found that 36% of students screened positive for an anxiety disorder and 20% screened positive for depression (Eisenberg et al. 2023). The reason that college may increase vulnerability for anxiety and depression may be due to increased stressors, such living independently for the first time, making major life decisions, financial stressors and greater academic responsibilities (Ibrahim et al. 2013; Moeller and Seehuus 2019; Pedrelli et al. 2015; Ramón‐Arbués et al. 2020). This trend may also be prevalent in students with disabilities. One study found that when comparing college students with and without disabilities, those with disabilities presented with higher rates of depression, anxiety and suicidality than peers without disabilities (Aguilar and Lipson 2021; Lipson et al. 2022). Research has estimated that 40% of adults with ID have at least one mental health disorder, which is twice the rates of psychopathology reported in the general population (Cooper et al. 2007; SAMHA 2023). This increased risk of psychopathology for adults with ID may be due to unique stressors related to ID, including stigma, unemployment and loneliness (Emerson 2011; Gilmore and Cuskelly 2014). Therefore, although there are limited data on mental health disorders among college students with ID, it is possible that these extant stressors for people with ID, in addition to the stressors of college, may increase their vulnerability to mental health problems.

Understanding the factors that predict mental health problems in college‐aged individuals is important to offer appropriate intervention to reduce the high rate of mental health disorders in this population. Concurrently, there has also been a great deal of work on factors that contribute to positive psychological functioning. Although positive psychology encompasses many topics (e.g., character strengths, resilience and thriving), one area closely related to mental health is life satisfaction, which is one of the subjective components of quality of life and involves the perception of one's life as a whole (Miller and Chan 2008; Schalock and Keith 1993, 2016). Among neurotypical college students, life satisfaction has been associated with improved mental health, including a negative relationship with both depression and anxiety (Guney et al. 2010; Ooi et al. 2022; Seo et al. 2018). Past research has posited that the relationship between depression and life satisfaction may be related to the core features of depression, particularly anhedonia, which decreases the ability for an individual to experience pleasure and is significantly associated with life engagement (McIntyre et al. 2022; Wong et al. 2024).

Research suggests that a major predictor of life satisfaction for the general population is a sense of belonging (Blau et al. 2016; Casagrande et al. 2020; Mellor et al. 2008; Pavot and Diener 1993). Belongingness, as a universal human need, is a motivational construct that enables individuals to create and maintain reciprocal social relationships characterised by attachment, companionship and support (Baumeister and Leary 1995). Belongingness can also act as a protective factor against mental health problems. Research has found that belongingness moderates the relationship between perceived stress and life satisfaction as well as the relationship between loneliness and depressive symptoms (Çivitci 2015; Zhou et al. 2023). Given that belongingness is an important predictor of life satisfaction and a protective factor against mental health disorders, it has been a target for intervention among historically marginalised college students (Walton and Cohen 2011).

Although there is no extant literature on belongingness among college students with ID, there is research that has suggested that young adults with ID experience a significant amount of loneliness. A recent study found that loneliness increased from adolescence to early adulthood for individuals with ID, which is different from the general population (for whom loneliness tends to be stable; Mund et al. 2020; Schiltz et al. 2024). This increase in loneliness may be associated with the transition out of high school, which has been described as ‘falling off a cliff’ for students with ID, partly due to the loss of services and supports provided through school but also the loss of the natural social community that school provides (Davies and Beamish 2009, 255).

### 1.1 Current Study

There is very little research on the psychological outcomes of participation in IPSE programmes for college students with ID. The current study aims to evaluate the psychological experiences of college students with ID, particularly the role of belongingness and mental health symptoms (i.e., anxiety and depressive symptoms, as the most prevalent mental health symptoms among college students) in predicting life satisfaction. This study will investigate whether these variables (life satisfaction, belongingness, anxiety and depressive symptoms) differ based on participant characteristics (e.g., year in the programme, gender and race) and programme characteristics (e.g., residential status and inclusive opportunities). Given past research among neurotypical college students has found a relationship between life satisfaction, belongingness and anxiety and/or depressive symptoms, understanding the relationship between these variables among college students with ID could help researchers and practitioners appropriately address mental health concerns and understand experiences of belongingness and how they contribute to positive mental health within this population.

## 2 Methods

### 2.1 Participants

Data were collected from 129 students and staff from 21 IPSE programmes across the United States. Inclusion criteria for student participants in this study were as follows: (a) have exited high school, (b) enrolled full‐time in an IPSE programme in the United States and (c) have ID. Student participants were identified as having ID via programme staff, according to the definition provided by the HEOA, which is as follows: (a) a student with significant limitations in both intellectual functioning and adaptive behaviour and (b) a student ‘who is currently, or was formerly, eligible for a free appropriate public education under the Individuals with Disabilities Education Act’ (HEOA 2008). Participants in this study may have co‐occurring disorders or disabilities (e.g., autism spectrum disorder, a mental health disorder) along with ID. Even though students in IPSE programmes may have co‐occurring disorders, most research on students in IPSE programmes still refer to them as having ID, and thus, this naming convention will be retained (Grigal et al. 2019).

The following inclusion criteria were set for IPSE programmes: (a) Curriculum is at least 2 years in length, (b) housed at a 4‐year college or university and (c) allow students to take coursework (either for credit or audit) with traditional college students.

### 2.2 Procedure

This study was approved by the Ohio State University Behavioral and Social Sciences Institutional Review Board (IRB). Study recruitment involved emailing IPSE programmes that met inclusion criteria. In total, 97 IPSE programmes were contacted with IRB‐approved email messages containing recruitment flyers to share with their students and staff. Individuals who were interested in the study clicked on a link on a recruitment flyer, which took them to a Qualtrics survey. Data were collected at one time point in fall 2024. During the period when the survey was open, 200 responses were received, but only 129 of those 200 responses were complete (i.e., finished the survey), meaning the survey had a 64.5% response rate. Of those 71 incomplete responses, 19 were from individuals who did not consent or assent to participate, and 52 were from individuals who started but did not finish the survey.

For students, the first page of the survey was a video of the researcher explaining the study and its relevant components. After watching this video, students could choose to leave the survey and come back (i.e., click the link again) after having their questions answered. If they chose to continue with the survey after having their questions answered, they engaged in an informed consent process. First, participants were asked to indicate if they were their own legal guardian, which would direct participants to the appropriate consent form. For example, for individuals without a legal guardian, they were directed to the individual consent form. For those with a legal guardian, they were directed to the parent or legal guardian consent form and then the individual assent form. For staff participants interested in the study, they clicked on a link on the recruitment flyer, which took them to the staff Qualtrics survey. For staff participants, the first page of their Qualtrics survey was a consent form. Like students, they could electronically sign the form with the option to download a copy for their records.

### 2.3 Measures

#### 2.3.1 Students

##### 2.3.1.1 Demographic Information

Demographic information was collected from students, including age, gender, race, ethnicity, residential status, social involvement at school, disability status and support completing the survey.

##### 2.3.1.2 Life Satisfaction

The *Quality of Life Questionnaire* (QOL.Q; Schalock and Keith 1993) is a 40‐item self‐report scale designed to measure quality of life in adults with ID. The QOL.Q has four subscales: (1) satisfaction, (2) competence/productivity, (3) empowerment/independence and (4) social belonging/community integration. Response options are on a 3‐point scale with verbal choices (Schalock and Keith 2004). Items from the satisfaction subscale were used in this study to measure life satisfaction, as has been done in a past study with adults with ID (Miller and Chan 2008). The satisfaction subscale consists of 10 items, each with three responses. Item scores are summed for a total score, ranging from 10 to 30 points, with higher scores indicating greater life satisfaction. Internal consistency in this study for the satisfaction subscale (calculated using ordinal alpha) was acceptable (α = 0.74), and Cronbach's alpha was in the questionable range (α = 0.63; Bland and Altman 1997).

##### 2.3.1.3 School Belongingness

To measure school belonging, we created a hybrid measure by including three items from the *National Survey of Student Engagement's* (NSSE) Sense of Belonging scale (Kuh 2001) and three items from the *Simple University Belonging Scale* (SUBS; Lingat et al. 2024). The NSSE has been used in a study comparing the experiences of college students with and without ID, although the sample of students with ID was underpowered and researchers could not examine the NSSE's psychometric properties (Hendrickson et al. 2015). Items on the NSSE's Sense of Belonging scale are rated on a 4‐point Likert scale (*strongly agree*, *agree*, *disagree*, *strongly disagree*). The NSSE's Sense of Belonging scale demonstrated good internal consistency (α = 0.85) (NSSE 2024). The belongingness scale used in the current study (using items from the NSSE and SUBS) demonstrated good internal consistency, calculated using ordinal alpha (α = 0.84) and acceptable internal consistency using Cronbach's alpha (α = 0.74).

Due to the limited information on the psychometric properties of the NSSE's Sense of Belonging scale, we also administered three items from the SUBS. The SUBS is a unidimensional measure of belonging designed for college students in online and face‐to‐face settings. It is a nine‐item scale with four response options (*NO!*, *no*, *yes*, *YES!*). The SUBS has previously been used with a small sample of college students with ID (Canis et al. 2023). In our study, the 4‐point Likert response options were accompanied by visual supports, which research has suggested increased comprehension of items for individuals with ID (Bell et al. 2018; Kooijmans et al. 2022). The combined belongingness scale used for this study used a sum score ranging from 4 to 24, with higher scores indicating a greater sense of belonging.

##### 2.3.1.4 Anxiety Symptoms

The *Glasgow Anxiety Scale for People with Intellectual Disability* (GAS‐ID; Mindham and Espie 2003) is a 27‐item self‐report scale that assesses anxiety symptoms in adults with ID. For each item, respondents endorse the frequency of experiencing that symptom, with response options on a 3‐point scale (0 = *never*, 1 = *sometimes*, 2 = *always*). The GAS‐ID also provides visual supports to enhance comprehension of items as well as the response options (e.g., a bar chart depicting the size difference between *never*, *sometimes* and *always*). Scores range from 0 to 52, with higher scores indicating more anxiety symptoms. The current study found good internal consistency for the GAS‐ID using both ordinal alpha (α = 0.84) and Cronbach's alpha (α = 0.80).

##### 2.3.1.5 Depressive Symptoms

The *Glasgow Depression Scale for People with a Learning Disability* (GDS‐LD; Cuthill et al. 2003) is a 20‐item self‐report scale that assesses depressive symptoms in adults with ID. Like the GAS‐ID, the response options on the GDS‐LD are on a 3‐point scale (0 = *never*, 1 = *sometimes*, 2 = *always*) and are accompanied by visual supports. Scores range from 0 to 40, with higher scores indicating more depressive symptoms. The current study found good internal consistency for the GDS‐LD using both ordinal alpha (α = 0.85) and acceptable internal consistency using Cronbach's alpha (α = 0.79).

#### 2.3.2 Programme Demographic Information

For staff members, programme demographic information was collected, including the number of years the programme had been in existence, the number of currently enrolled students, credentialing (offers a credential, does not offer a credential), the level of academic inclusion, the use of peer mentors from the larger university, social inclusion (e.g., residing in campus housing, eating in the campus dining hall, access to extracurricular activities and attending social events on campus; Grigal et al. 2012) and admissions criteria.

### 2.4 Data Analysis

To compare life satisfaction, belongingness, anxiety symptoms and depressive symptoms across groups based on demographic variables, a series of independent‐samples *t*‐tests were performed.

To examine the relationship among life satisfaction, belongingness, anxiety symptoms and depressive symptoms, a hierarchical regression analysis was performed. In a hierarchical regression, each independent variable is entered in a specific order, which allows for examination of the influence of each independent variable. Using a hierarchical regression analysis tests the overall model, but also the unique effect of adding independent variables at each step. In the first step of the hierarchical regression, race was added as a control variable, because prior published research has suggested that race and belongingness are significantly related (Lee and Shapiro 2023; Walton and Cohen 2007). In the second step, anxiety symptoms were added. In the third step, depressive symptoms were added, given the previously established strong relationship between depression and life satisfaction (Ooi et al. 2022). Finally, in the fourth step, belongingness was added to assess the additional impact of belongingness on life satisfaction, over and above mental health symptoms. At each step, the change in *R* ^*2*^ was noted.

To test for moderators in the relationship among life satisfaction, belongingness, anxiety symptoms and depressive symptoms, interaction terms were created using mean‐centred variables (anxiety*belongingness, depression*belongingness). The hierarchical regression was run again including each mean‐centred variable as a predictor to test for additive effects. Then, in the final step of the hierarchical regression, the interaction term was entered to see if the interaction accounts for additional variance in the dependent variable (i.e., life satisfaction).

Descriptive statistics, group mean comparisons and regression analyses were conducted using SPSS Version 29.0 (IBM Corp 2022). Internal consistencies for scales were conducted using R Version 4.3.3 using packages *psych*, *GPArotation* and *Rcmdr* (Bernaards and Jennrich 2005; Fox 2017; R Core Team 2021; Revelle 2024).

#### 2.4.1 Internal Reliability

To examine the internal reliability of the scales used in this study, ordinal alpha was used (Zumbo et al. 2007). Both ordinal alpha and Cronbach's alpha measure the internal consistency of a scale, but ordinal alpha uses a polychoric correlation matrix, rather than a Pearson covariance matrix, to calculate reliability. If data are non‐continuous (e.g., items that have a Likert‐type response format), using a Pearson covariance matrix can distort reliability estimates, often underestimating alpha (Gadermann et al. 2019). Therefore, calculating alpha using a polychoric correlation matrix may provide a more accurate reliability estimate for ordinal data.

## 3 Results

### 3.1 Student Demographics

Students ranged in age from 18 to 31 years, with a mean age of 21.87 years (SD = 2.5). Sixty‐one per cent (*n* = 79) of student participants identified as male, 38.0% (*n* = 49) identified as female, and 0.8% (*n* = 1) identified as non‐binary. The distribution of student participants by race consisted of 69.0% White (*n* = 89), 14.0% Black or African American (*n* = 18), 7.8% Asian (*n* = 10), 7.8% Multiracial (*n* = 10) and 1.6% American Indian or Alaska Native or Native Hawaiian or Pacific Islander (*n* = 2). A small number of students (*n* = 8) identified as not having a disability, despite the fact that having a disability is a requirement of being in an IPSE programme.

In terms of mental health symptoms, students scored higher on anxiety symptoms than on depressive symptoms, although students were, on average, above the cut‐off scores for both anxiety and depression. The overall participant mean score on the GDS‐LD was 13.1 (SD = 5.7), which has a cut‐off score of 13 (indicating a possible depressive disorder). Fifty‐two per cent (*n* = 67) of the sample scored 13 or greater on the GDS‐LD. The overall participant mean score on the GAS‐ID was 20.9 (SD = 2.6), which has a cut‐off score of 17 (indicating a possible anxiety disorder). Seventy‐one per cent (*n* = 92) of the sample scored 17 or greater on the GAS‐ID. Forty‐six per cent (*n* = 59) of the sample scored above cut‐off on both the GDS‐LD and the GAS‐ID.

### 3.2 Programme Demographics

The 21 IPSE programmes varied in terms of their inclusive policies related to academics, housing and social involvement for the students with ID. In terms of academic inclusion (i.e., students in the IPSE programme took classes with degree‐seeking students), the majority of programmes (*n* = 11) reported that 50%–99% of college classes were inclusive, with six programmes (28.6%) reporting that 100% of their college classes were inclusive. In terms of housing and campus support, 81% (*n* = 17) of programmes reported that they provided on‐campus housing options to students. Most programmes (*n* = 15) also reported that their students could access general mental health services at their college, leaving nearly a quarter (23.8%) of programmes where students in the IPSE programme only had access to programme‐specific mental health services, if available. See Tables 1 and 2 for characteristics of student participants and IPSE programmes.

**TABLE 1:** Demographic characteristics of student participants (*n* = 129).

	*N* (%)
Gender	
Female	79 (61.2)
Male	49 (38.0)
Non‐binary/third gender	1 (0.8)
Race	
White	89 (69.0)
Asian	10 (7.8)
Black	18 (14.0)
American Indian or Alaska Native or Native Hawaiian or Pacific Islander	2 (1.6)
Multiracial	10 (7.8)
Ethnicity	
Non‐Hispanic	111 (86.0)
Hispanic	18 (14.0)
Residential status	
On‐campus in a dorm or residence hall	50 (38.8)
Off‐campus living with roommates or alone	55 (42.6)
Off‐campus living with family or caretakers	24 (18.6)
Eat meals in the dining hall at college or university	
Yes	78 (60.5)
No	46 (35.7)
Has joined extracurricular activities	
Yes	81 (62.8)
No	48 (37.2)
First year at school	
Yes	66 (51.2)
No	63 (48.8)
Self‐identifies as having a disability	
Yes	121 (93.8)
No	8 (6.2)
Received support to complete survey (e.g., family member or support person)	
Yes	67 (51.9)
No	52 (48.1)

**TABLE 2:** Characteristics of IPSE programmes (*n* = 21).

	*N* (%)
Type of institution	
Public	18 (85.7)
Private	3 (14.3)
Geographic region	
Northeast	2 (9.5)
South	10 (47.6)
West	5 (23.8)
Midwest	4 (19.0)
IPSE programme has CTP status	
Yes	17 (81.0)
No	4 (19.0)
IPSE programme has received a TPSID grant	
Yes	9 (42.9)
No	11 (52.4)
Not sure	1 (4.8)
IPSE programme is currently under TPSID grant	
Yes	5 (23.8)
No	16 (76.2)
Access to campus mental health services	
Students have access to general mental health services at the college or university	15 (71.4)
Students have access to programme‐specific mental health services	5 (23.8)
Unsure of access to mental health services	1 (4.8)
Percentage of classes students take with students not in their programme (i.e., percentage of inclusive classes)	
100%	6 (28.6)
50%–99%	11 (52.4)
< 50%	4 (19.0)
Programme has peer mentors (i.e., neurotypical students enrolled at college or university that provide support to students in your programme)	
Yes	21 (100.0)
No	0 (0.0)
Programme has students who do not meet criteria for ID	
Yes	4 (19.0)
No, all have ID	17 (81.0)

Independent samples *t*‐tests were conducted to test for group differences in belongingness, life satisfaction, anxiety symptoms and depressive symptoms across demographic variables. There was a significant difference in anxiety symptoms between men and women, *t*(126) = −2.07, *p* < 0.05, with women reporting more anxiety symptoms (*M* = 22.6, SD = 8.2) than men (*M* = 19.9; SD = 6.5). Hispanic participants reported higher levels of anxiety (*M* = 25.1, SD = 7.3) and lower life satisfaction (*M* = 23.2, SD = 3.7) than non‐Hispanic participants (for anxiety: *M* = 20.2, SD = 7.2; for life satisfaction: *M* = 24.7, SD = 2.8). Participants who identified as having a disability also endorsed higher levels of anxiety (*M* = 21.2, SD = 7.1) and higher levels of belongingness (*M* = 20.7, SD = 2.5) than those who did not self‐report having a disability (for anxiety: *M* = 15.8, SD = 9.3; for belongingness: *M* = 18.4, SD = 3.3). Finally, those who lived with roommates or alone (in either on‐ or off‐campus housing) reported greater life satisfaction (*M* = 24.7, SD = 2.9) than those who lived with family members or caretakers, *t*(127) = −2.10, *p* < 0.05.

There were no significant differences in belongingness, anxiety symptoms, depressive symptoms or life satisfaction in terms of race, level of social involvement at school (e.g., attending social events, being part of extracurricular activities, eating in the dining hall) or being a first‐year student. However, an independent samples *t*‐test found that individuals in programmes without access to general mental health services reported more anxiety symptoms (*M* = 23.7, SD = 6.9) than those in programmes with access to general mental health services (*M* = 20.3, SD = 7.4).

A hierarchical regression was performed to understand the relationship between belongingness, anxiety symptoms, depressive symptoms and life satisfaction. Step 1 of the analysis indicated a significant relationship, with race, ethnicity and residential status accounting for 7.2% of the variance in life satisfaction, *F*(3, 125) = 3.22, *p* = 0.025. Of the three variables, only residential status explained unique variance in life satisfaction ( β = 18; *t* = 2.06, *p* = 0.042).

In Step 2, anxiety symptoms were added as an independent variable. Results showed that the second model was significant, *F*(4, 124) = 4.47, *p* = 0.002. Anxiety symptoms were significantly associated with life satisfaction ( β = − 0.24, *t* = −2.77, *p* = 0.006). In Step 3, depressive symptoms were added to the model. Depressive symptoms were significantly associated with life satisfaction ( β = − 0.39; *t* = −3.86, *p* < 0.001). Finally, in Step 4, belongingness was added to the model. The model was significant, *F*(6, 122) = 10.73, *p* < 0.001, and explained 34.5% of the variance in life satisfaction. This model was a significant improvement from Step 3 and accounted for 12.5% additional variance in life satisfaction, above and beyond the variance accounted for by anxiety and depressive symptoms, when controlling for race, ethnicity and residential status. In the final model, only depressive symptoms and belongingness were significantly associated with life satisfaction ( β = − 0.32; *t* = −3.42, *p* < 0.001; β = 0.36; *t* = 4.83, *p* < 0.001, respectively).

To test for moderators in the relationship between belongingness, anxiety symptoms, depressive symptoms and life satisfaction, interaction terms were created. In Step 5 of the regression, the model from Step 4 was run again (residential status, anxiety symptoms, depressive symptoms and belongingness) with the interaction terms (anxiety*belongingness and depression*belongingness). The overall model was significant, *F*(8, 120) = 8.25, *p* < 0.001. However, neither anxiety*belongingness nor depression*belongingness was significantly associated with life satisfaction, indicating that moderation is not present in this relationship. See Table 3 for a summary of the results of the hierarchical regression analyses.

**TABLE 3:** Hierarchical regression analyses for predicting life satisfaction.

	*R* ^2^	∆ *R* ^2^	*B*	β	*p*
Step 1	0.07	0.07			0.025*
Race			−0.82	−0.13	0.15
Ethnicity			−1.45	−0.17	0.06
Residential status					0.04*
Step 2	0.13	0.05			0.006**
Race			−0.80	−0.13	0.15
Ethnicity			−0.98	−0.12	0.19
Residential status			1.34	0.18	0.04*
Anxiety symptoms			−0.10	−0.24	0.006**
Step 3	0.22	0.09			< 0.001***
Race			−0.77	−0.12	0.15
Ethnicity			−0.87	−0.10	0.23
Residential status			1.37	0.18	0.03*
Anxiety symptoms			−0.003	−0.01	0.94
Depressive symptoms			−0.20	−0.39	< 0.001***
Step 4	0.35	0.13			< 0.001***
Race			−0.73	−0.11	0.14
Ethnicity			−0.88	−0.10	0.18
Residential status			0.95	0.13	0.10
Anxiety symptoms			−0.004	−0.01	0.93
Depressive symptoms			−0.17	−0.32	< 0.001***
School belongingness			0.42	0.36	< 0.001***
Step 5	0.36	0.01			0.370
Race			−0.71	−0.11	0.15
Ethnicity			−0.84	−0.10	0.21
Residential status			0.98	0.13	0.09
Anxiety symptoms			0.01	0.02	0.82
Depressive symptoms			−0.19	−0.37	< 0.001***
School belongingness			0.43	0.38	< 0.001***
Anxiety symptoms*School belongingness			0.01	0.06	0.53
Depressive symptoms*School belongingness			−0.02	−0.13	0.20

* *p* < 0.05.

** *p* < 0.01.

*** *p* < 0.001.

## 4 Discussion

There were significant differences in life satisfaction and level of anxiety based on gender, residential status and access to mental health services. Furthermore, when examining the relationships between mental health symptoms, belongingness and life satisfaction, belongingness accounted for an additional 12.5% of variance in life satisfaction, above and beyond the variance accounted for by mental health symptoms.

In terms of mental health symptoms, students in the current study reported high levels of depressive and anxiety symptoms, with nearly half the students (46%) scoring above cut‐off on both the GDS‐LD and GAS‐ID, indicating the possible presence of depressive and anxiety disorders. It is unsurprising that students who presented with depressive symptoms would also be likely to present with anxiety symptoms, as depression and anxiety are highly comorbid, especially among people with ID (Cooper et al. 2007; Einfeld et al. 2011). In the current study, anxiety symptoms among college students with ID were particularly high (71% of students scored above cut‐off for an anxiety disorder), much higher than indicated in other population‐based samples of adults with ID, where the prevalence has been reported between 3.8% and 5.5% (Cooper et al. 2007; Mazza et al. 2020). Although not as prevalent, depressive symptoms were also high in the study sample (52% of the students scored above cut‐off for a depressive disorder) and were higher than estimates in population‐based samples (4.1%–6.7%; Cooper et al. 2007; Mazza et al. 2020). The relatively high levels of anxiety and depressive symptoms experienced by college students with ID may be due to the confluence of stressors associated with college as well as stressors associated with having ID.

Results of the current study also indicated differences in life satisfaction and mental health symptoms of college students with ID based on programme‐related characteristics. Students who lived with roommates or alone (in on‐ or off‐campus housing) had significantly higher levels of life satisfaction than students who lived with family or caregivers. Living independently away from family is a normative activity for neurotypical young adults and signifies an important developmental milestone in emerging adulthood (Benson and Furstenberg 2006; Sharon 2016). For adults with ID, who have a documented history of lack of self‐determination and choices over where and with whom they live, independent living seems to be particularly important for their life satisfaction in college, as has been found in studies among adults with ID in community settings (Stancliffe et al. 2009).

Additionally, students in programmes with access to mental health services at their college (i.e., a university counselling centre or UCC) reported lower levels of anxiety than students without access to a UCC. Although the directionality of this relationship cannot be determined, research has suggested that adults with ID experience health inequities regarding their access to mental health treatment compared to their neurotypical peers, often due to a lack of training among available providers and mistaken beliefs that people with ID either do not experience mental health problems or they cannot benefit from psychotherapy (Dell'Armo and Tassé 2024; Einfeld et al. 2011; McCarthy and Boyd 2002; Whittle et al. 2018). A recent study of 33 IPSE programme directors similarly found that despite the presence of significant mental health concerns among their students, only one‐third of these programmes referred students with ID to their UCC, due to ineligibility for services (e.g., not paying requisite university fees and ineligible due to ID diagnosis), long waitlists or no mental health services available at their college (McKnight‐Lizotte et al. 2021).

### 4.1 Limitations

There are a few limitations to the current study. Firstly, students may have had co‐occurring diagnoses, including autism spectrum disorder (ASD; Grigal et al. 2019). Although students were not directly asked whether they had another diagnosis besides ID, it is possible that students in the current study may have had ID and ASD and that ASD may have acted as a confounding variable in the rates of psychopathology reported. Particularly, the high levels of anxiety found in our sample could be due to an over‐representation of ASD, as anxiety and ASD are highly comorbid (Buck et al. 2014; Hollocks et al. 2019). Secondly, half of the students (51.9%) reported receiving support in completing the survey, such as from a family member or support person. Given that many of the questions in this survey were about psychological experiences, the students might have responded to these questions in a more socially desirable way (Finlay and Lyons 2002; Perry 2004) because of the presence of this support person. Because the survey was completed online, it is impossible to know the extent of support provided for students and how that may have influenced students' responses. Finally, the measure of belonging used in this study had not been previously validated among a large sample of college students with ID. Therefore, it is possible that the measure was not tapping into the latent construct of belonging for college students with ID. Future studies should investigate the adaptation of existing belonging measures to allow for appropriate comparison between people with and without ID.

### 4.2 Implications for Researchers and Practitioners

There are a few implications of this work on research and practice. Firstly, future studies should consider measuring these variables over a longer period of time (e.g., 6–12 months) to examine changes over time, as has been done in past studies with neurotypical college students (Costello et al. 2022; Hausmann et al. 2007; Marksteiner et al. 2019). Furthermore, given recent research on daily fluctuations of belongingness, future research should measure belongingness more frequently and across contexts (e.g., within programme activities, with peer mentors, at university‐wide events) to understand whether belongingness among college students with ID follows similar patterns to neurotypical students (Dutcher et al. 2022; Ruedas‐Gracia et al. 2023). Given the relationship between access to mental health services and anxiety, future research should examine treatment access and outcomes for college students with ID and co‐occurring mental health disorders. Understanding who is seeking mental health treatment, their ability to access treatment and the types of treatments available to students will be helpful information in bolstering mental health support for this population of college students, given their vulnerability to psychopathology and barriers to accessing psychotherapy (Mazza et al. 2020; Whittle et al. 2018).

Relatedly, although this study found that students with ID experience anxiety and depressive symptoms at similar rates to neurotypical college peers, they do not have access to the same mental health supports. Practitioners should consider policy changes that would increase access to mental health support for this population, including training mental health clinicians in working with individuals with ID and increased mental health screenings within IPSE programmes to identify students who may be experiencing significant mental health concerns.

## Funding

This work was supported in part with funding from the Nisonger Center Research Fund.

## Ethics Statement

Research was undertaken with the understanding and written consent of each participant and in accordance with ethical principles. This study was independently reviewed and approved by the Ohio State University Institutional Review Board (#2023B0255).

## Conflicts of Interest

The authors declare no conflicts of interest.

## Author Note

This study was submitted in partial fulfillment of the Doctor of Philosophy requirements at The Ohio State University for the first author.

## Data Availability

The data that support the findings of this study are available from the corresponding author upon reasonable request.
